# Recombinant Invasive *Lactococcus lactis* Carrying a DNA Vaccine Coding the Ag85A Antigen Increases INF-γ, IL-6, and TNF-α Cytokines after Intranasal Immunization

**DOI:** 10.3389/fmicb.2017.01263

**Published:** 2017-07-11

**Authors:** Pamela Mancha-Agresti, Camila Prosperi de Castro, Janete S. C. dos Santos, Maíra A. Araujo, Vanessa B. Pereira, Jean G. LeBlanc, Sophie Y. Leclercq, Vasco Azevedo

**Affiliations:** ^1^Laboratory of Cellular and Molecular Genetics, Department of General Biology, Instituto de Ciências Biológicas – Universidade Federal de Minas Gerais Belo Horizonte, Brazil; ^2^Laboratório de Inovação Biotecnológica, Fundação Ezequiel Dias Belo Horizonte, Brazil; ^3^Centro de Referencia para Lactobacilos – Consejo Nacional de Investigaciones Científicas y Técnicas San Miguel de Tucumán, Argentina

**Keywords:** lactic acid bacteria, *Lactococcus lactis*, tuberculosis, Ag85A, mucosal vaccine

## Abstract

Tuberculosis (TB) remains a major threat throughout the world and in 2015 it caused the death of 1.4 million people. The Bacillus Calmette-Guérin is the only existing vaccine against this ancient disease; however, it does not provide complete protection in adults. New vaccines against TB are eminently a global priority. The use of bacteria as vehicles for delivery of vaccine plasmids is a promising vaccination strategy. In this study, we evaluated the use of, an engineered invasive *Lactococcus lactis* (expressing Fibronectin-Binding Protein A from *Staphylococcus aureus*) for the delivery of DNA plasmid to host cells, especially to the mucosal site as a new DNA vaccine against tuberculosis. One of the major antigens documented that offers protective responses against *Mycobacterium tuberculosis* is the Ag85A. *L. lactis* FnBPA^+^ (pValac:*Ag85A)* which was obtained and used for intranasal immunization of C57BL/6 mice and the immune response profile was evaluated. In this study we observed that this strain was able to produce significant increases in the amount of pro-inflammatory cytokines (IFN-γ, TNF-α, and IL-6) in the stimulated spleen cell supernatants, showing a systemic T helper 1 (Th1) cell response. Antibody production (IgG and sIgA anti-Ag85A) was also significantly increased in bronchoalveolar lavage, as well as in the serum of mice. In summary, these findings open new perspectives in the area of mucosal DNA vaccine, against specific pathogens using a Lactic Acid Bacteria such as *L. lactis.*

## Introduction

Tuberculosis (TB) is a disease with a high incidence rates around the world that was directly responsible for the death of about 1.4 million people in 2015 and an additional 0.4 million among people co-infected with HIV (WHO, 2016).

Bacillus Calmette-Guérin (BCG) is an attenuated form of *Mycobacterium bovis* that is the only licensed vaccine in use, which prevents TB in infants and in the disseminated disease. Nevertheless, it has limitations to protect adolescents and adults against the pulmonary disease (Kaufmann, 2010; da Costa et al., 2015). Another bottleneck of this vaccine is the memory immunity generated which declines with the age (Vijayalakshmi et al., 1993; Kaufmann, 2004). A new vaccine against TB, especially for a pulmonary disease is eminently needed.

It is well known that the main protective antigens of *M. tuberculosis* are secreted proteins of the culture filtrate. Among these proteins Ag85A, B and C, which belong to the antigen 85 (Ag85) complex, have been reported to provide protection against TB (D’Souza et al., 2003; Jain et al., 2008). They are considered virulence factors with high immunogenicity (Armitige et al., 2000; Dietrich et al., 2006). The Ag85A is an immunodominant protein (Huygen et al., 1996) able to increase the Th1 (T helper cells type 1) cytokine response (Romano et al., 2006). Furthermore, Ag85A stimulates IFN-γ production by peripheral blood mononuclear cells (PBMC) from both BCG-vaccinated (Anuradha et al., 2007, 2008) and TB patients (Priya et al., 2009).

The pro-inflammatory cytokine IFN-γ and TNF-α are produced by CD4^+^ Th-1 cells, which are powerfully related with protection against *M. tuberculosis* infection, and stimulate the development of a T-helper (Th) 1 T cell response. The intracellular microbicidal activities of alveolar macrophages could be increased by the synergization of theses cytokines to activate macrophages promoting the induction of nitric oxide synthase (NOS_2_) which participates in killing of *M. tuberculosis* (Saito and Nakano, 1996; Lighvani et al., 2001). The Th-1 immune response is enough to keep the microorganism inside the granuloma and even more, avoid further spread (Ottenhoff, 2012).

DNA vaccination is an effective strategy to introduce genetic material into the host cells. It is able to induce long-lasting humoral and cell-mediated immune responses against the encoded antigens (Donnelly et al., 1996). This approach often uses attenuated enteroinvasive bacteria for its delivery (Schoen et al., 2004; Detmer and Glenting, 2006), which can present a risk since the reversion to their virulent phenotype through mutation or gene transfer is possible (Dunham, 2002).

The use of non-pathogenic bacteria, such as lactic acid bacteria (LAB), as a DNA delivery vehicle to the mucosal surface, could be a better and safer alternative for this purpose (Wells and Mercenier, 2008; Rosales-Mendoza et al., 2016). LAB, especially lactobacilli, and *Lactococcus lactis*, are used in the food industry and are classified as “**G**enerally **R**egarded **A**s **S**afe” (GRAS) organisms due to their lack of pathogenicity, plus they have the criteria of the competent **Q**ualified **P**resumption of **S**afety (QPS) according to the European Food Safety Authority (EFSA).

*Lactococcus lactis* is the best-characterized member of the LAB group being considered its model organism and has been used for the production and delivery of antigens and cytokines (Bermúdez-Humarán et al., 2005; Cortes-Perez et al., 2007; Hugentobler et al., 2012; Robert and Steidler, 2014) as well as a vehicles to delivery DNA (Wells and Mercenier, 2008).

The key to achieving an efficient delivery of DNA to eukaryotic cells is the internalization of the bacterial carrier (Grillot-Courvalin et al., 1999) mediated by cell-specific surface receptors and invasins from bacteria. Thus, to improve the DNA delivery with LAB, into mammalian cells, invasin-encoding genes such as the Fibronectin-binding protein A (FnBPA) from *Staphylococcus aureus* (Que et al., 2001) have been expressed in *L. lactis*. The invasive power of recombinant *L. lactis* FnBpA was evaluated by the transformation of pValac:*gfp* vector (Innocentin et al., 2009); and this gfp-coding plasmid was able to encroach Caco-2 cells, and the epithelial cells have shown an increased GFP expression when compared to the wild-type *L. lactis* (Innocentin et al., 2009) indicating the potential of recombinant *L. lactis* for gene delivery.

In this study, our aim was to construct the invasive *L. lactis* FnBPA^+^ expressing the Ag85A TB antigen (pValac:*Ag85A*) strain and evaluate its immunological response in a rodent model.

## Materials and Methods

### Bacterial Strains and Plasmids

The bacterial strains and plasmids used in this work are listed in **Table 1**. *Escherichia coli* TG1 and *E. coli* TOP 10 were grown aerobically in Luria-Bertani (Acumedia) medium (LB) at 37°C with shaking. *L. lactis* FnBPA^+^(Que et al., 2001) was grown in M17 (Difco, Sparks, MD, United States) medium supplemented with 0.5% glucose (GM17) at 30°C without shaking. When required, recombinant bacteria were selected by addition of antibiotics: for *L. lactis* FnBPA^+^(pValac:*Ag85A*) erythromycin (Ery, Sigma–Aldrich) at 5 μg/mL and chloramphenicol (Cm, Sigma–Aldrich) at 5 μg/mL; for *E. coli* (pValac:*Ag85A*), Cm at 10 μg/mL were used.

**Table 1:** Bacterial strains and plasmids used in this work.

Bacterial strain and plasmids	Characteristics	Reference
*Escherichia coli* TG1	*E. coli* K-12-derived strain; F′ [*traD36 proAB*^+^ lacI^q^ *lacZ***Δ***M15*] supE *thi-1* **Δ**(*lac-proAB*) **Δ***(mcrB*-*hsdSM*)5, (rK**^-^**mK**^-^**)	Lucigen
*Escherichia coli* TG1 pValac:*gfp*	*E. coli* TG1 strain carrying the pValac*:gfp* plasmid	Guimarães et al., 2009
*Escherichia coli* TOP10	*E. coli* K-12-derived strain; F-mcrA **Δ**(mrr-hsdRMS-mcrBC) **Φ** 80lacZ**Δ**M15 **Δ**lacX74 nupG recA1 araD139 **Δ**(ara-leu)7697 galE15 galK16 rpsL(Str^R^) endA1 aaaa^-^	Invitrogen
*Lactococcus lactis* MG1363 FnBPA^+^	*L. lactis* MG1363 strain expressing FnBPA of *Staphylococcus aureus*	(Que et al., 2001)
*Lactococcus lactis* MG1363 FnBPA^+^ (pValac:*Ag85A*)	*L. lactis* MG1363 strain expressing FnBPA of *S. aureus* carrying the: pValac:*Ag85A* plasmid	This work

**Plasmids**	**Characteristics**	**Reference**

Zero Blunt^®^ TOPO^®^	Cloning vector (Km^R^, ccdB gene fused to the C-terminus of the LacZα fragment)	Invitrogen
pValac vector	Eukaryotic expression vector (pCMV/Cm^r^/RepA/RepC)	Guimarães et al., 2009
pValac:*gfp*	pValac vector containing *gfp* ORF	Guimarães et al., 2009
pValac:*Ag85A*	pValac vector coding *Ag85A* ORF	This work

*Ag85A, protein of *Mycobacterium tuberculosis; pValac* vaccination using lactic acid bacteria, *pCMV* cytomegalovirus promoter, *RepA* and *RepC* replication origins, *Cm^*r*^* chloramphenicol resistance gene, *gfp* green fluorescent protein coding sequence, *Km^*R*^* kanamicine resistance gene.*

### DNA Manipulations

Plasmid DNA from *E. coli* and *L. lactis* were isolated as described above (Green and Sambrook, 2012) with some modifications: for *L. lactis*, TES (25% sucrose, 1 mM EDTA, 50 mM Tris–HCl, pH 8) containing lysozyme (Sigma–Aldrich) (10 mg/mL) was added to the samples for 1 h at 37°C to prepare protoplasts. Electroporation of *L. lactis* was also performed as previously described (Langella et al., 1993) *E. coli* transformants were plated onto LB agar plates containing the required antibiotic for 24 h at 37°C, whereas GM17 have been used for *L. lactis* transformants with the required antibiotic and were counted after 24 h of incubation at 30°C.

### pValac: *Ag85A* and *L. lactis* FnBPA^+^ (pValac:*Ag85A*) Construction

The entire Ag85A open reading frame (ORF) from the genomic DNA of *M. tuberculosis* H37Rv strain was amplified by PCR using the Pfx Platinum^®^ High-Fidelity DNA Polymerase (Invitrogen). The individual oligonucleotide sequences used for Ag85A: 5′-*GGATCC****ACCATGG***AGCTTGTTGACAGGGTTCG-3′ (forward) and 5′-*GAATTC*CTAGGCGCCCTGGGGCGC-3′ (reverse), were constructed with the artificial restriction sites of *Bam*HI and *Eco*RI, respectively, and the customized Kozak sequence. The PCR product was isolated from agarose gels using a commercial kit (Kit illustra^TM^ GFX^TM^ PCR DNA). The amplified product was then cloned into the Zero Blunt^®^ TOPO^®^ vector (Invitrogen), and transformed into *E. coli* Top10 cells as described by the manufacturer. The intermediate plasmid, pTopo:*Ag85A*, was cleaved with *Bam*HI and *Eco*RI, and the Ag85A fragment was subcloned into the pValac vector (Guimarães et al., 2009) previously cleaved with the same restriction enzymes, agarose gel purified, ligated using the T4 DNA ligase (Invitrogen) and transformed into *E. coli* TG1 generating the *E. coli* TG1 (pValac:*Ag85A*) strain. The insert integrity was confirmed by DNA sequence analysis, using the BigDye Terminator v3.1 Cycle Sequencing Kit (Applied Biosystems) and the ABI3130 sequencing equipment. Finally, the pValac:*Ag85A* was transformed into *L. lactis* FnBPA^+^ strain, generating the *L. lactis* FnBPA^+^ (pValac:*Ag85A*) strain used in this study. This construction was confirmed by PCR using specific primers for the pValac vector and primers of fibronectin ORF.

### Ag85A Production by Eukaryotic Cells

The pValac:*Ag85A* plasmid was assayed for Ag85A expression by transfection into the Chinese hamster ovarian cell line [Flp-In^TM^-CHO (Invitrogen)] (CRL 12023)-ATCC. For this purpose, CHO cells were cultured in complete Nutrient Mixture F12 Ham media, supplemented with 10% fetal bovine serum, 1% L-glutamine, zeocin (100 ng/mL) and 2.5% HEPES. At about 90–95% of confluence, CHO cells were transfected with 4 μg of pValac:*Ag85A* vector or no plasmid (negative control) using Lipofectamine^TM^ 2000 (Invitrogen), as described by the supplier. The protein expression was assessed by confocal microscopy and flow cytometry. The pValac:*gfp* was used to standardize the transfection experiment. The cells producing the GFP permitted visualization of the transfection efficiency. After 48 h of transfection, pValac:*Ag85A*-transfected cells, as well as the control cells, were fixed with paraformaldehyde (Sigma–Aldrich) 4% for 15 min and permeabilized with 0.1% Triton X-100 for 10 min. The cells were incubated for 1 h with murine monoclonal anti-Ag85A antibody (H-2b haplotype mice – Mab DT-17/4, source: Professor Kris Huygens, Pasteur Institute of Brussels, Brussels, Belgium) diluted 1/20 in PBS/BSA 1% (*Bovine Serum Albumin*) (Drowart et al., 1992; Huygen et al., 1994). The CHO cells were then incubated with secondary goat anti-mouse IgG (H+L) Cross-Adsorbed, Alexa Fluor^®^ 488 (Invitrogen, 4 μg/mL, diluted 1/500) and with DAPI (4,6′-diamidino-2-phenylindole, Invitrogen, 2 μg/mL), for cell nucleus staining, for 1 h in reduced light conditions. The samples were mounted with hydramount and the images were captured using Zeiss LSM 510 META inverted confocal laser-scanning microscope. Images were collected and analyzed using Zeiss LSM Image Browser software. Cells transfected with the pValac:*gfp* plasmid was used as positive control.

For flow cytometry analysis, the pValac:*Ag85A*-transfected cells and control of non-transfected cells, as well as cells transfected with pValac:*gfp* were evaluated. For this purpose, 10^6^ cells were fixed and permeabilized using a Staining Buffer Set kit (eBioscience). Transfected cells with pValac:*Ag85A* were then incubated with murine monoclonal anti-Ag85A (MAb-DT-17/4) antibody for 30 min and then incubated with secondary goat anti-mouse IgG (H+L) Cross-Adsorbed, Alexa Fluor^®^ 488 (Invitrogen, 4 μg/mL, diluted 1/500) for 30 min. Afterward, the cells were fixed with paraformaldehyde and quantification of Ag85A producing CHO cells was performed by the use of the FACScan (Becton Dickinson Bioscience) equipment. The acquired data were analyzed using the FlowJo program (TreeStar, Ashland, OR, United States).

### Mice

Conventional female C57BL/6 inbred mice, 4–6 weeks of age were obtained from Centro de Bioterismo (CEBIO) of the Universidade Federal de Minas Gerais (UFMG, Brazil). Procedures and manipulation of animals followed the rules of the Ethical Principles in Animal Experimentation, accepted by the Ethics Committee on Animal Experiments (CETEA/UFMG/Brazil). All animals were maintained in collective cages (5 animals/group) in an environmentally controlled room with a 12-h light/dark cycle and given free access to water and food.

### Immunization Assay with *L. lactis* FnBPA^+^ (pValac:*Ag85A*)

**FIGURE 1:**
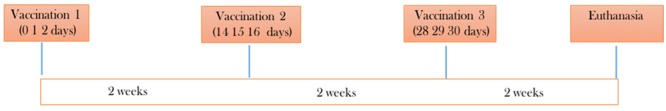
Protocol of immunization: For vaccination study mice were vaccinated three times at 2 weeks interval. On day 42 from the start of immunization, all animals were euthanized.

C57BL/6 mice were randomly branched into the following experimental groups: saline (negative control), *L. lactis* FnBPA^+^ (invasive *L. lactis* strain, negative control), *L. lactis* FnBPA^+^ (pValac:empty), and *L. lactis* FnBPA^+^ (pValac:*Ag85A*). Each animal was immunized by intranasal route with 20 μl (10 μl in each nostril) containing 2 × 10^9^ colony forming units (CFU). The immunizations were administrated at three different time points (days 0, 14, and 28) (Bermúdez-Humarán et al., 2003) and at each stage, the mice were immunized for three consecutive days. On day 42 from the start of immunization, all animals were anesthetized by intraperitoneal injection with a ketamine and xylazine 16 mg/kg mixture (Agener União) (**Figure 1**). Blood samples were collected from the inferior vena cava. In this study two individual experiments were performed, five animals were used in each group for each individual protocol and each animal was analyzed individually.

### Characterization of the Systemic Cellular Immune Response Profile

Cytokine production was assessed in culture supernatants of stimulated splenocytes to evaluate the systemic cellular immune response profile generated by administration of *L. lactis* FnBPA^+^(pValac:*Ag85A*) strain. Briefly, spleens were removed on the day of sacrifice, macerated and red blood cells from spleens were lysed. 1 × 10^6^ cells/spleen from each immunized animal, in triplicate, were added to wells of a microplate containing RPMI-1640 (Sigma–Aldrich), medium supplemented with 10% fetal bovine serum (Gibco), sodium pyruvate 1 mM, non-essential amino acids 1 mM (MEM NEAA—Gibco), gentamicin 25 μg/mL(Gibco), and L-glutamine 2mM (LGC Biotecnologia). The cells were incubated with medium alone to the negative control (non-stimulated cells) or in medium containing 5 μg/mL of Ag85A recombinant protein (ABCAM Catalog Number: P0A4V2). Concanavalin A (ConA) 16 μg/mL, was used as a positive control. Cells were incubated at 37°C in an atmosphere of 5% CO2. After 48 h of stimulation, the cell-free supernatants were harvested to measure the cytokines profile. A cytometric bead array (CBA; mouse Th1/Th2/Th17 cytokine kit; BD Biosciences) was used according to the manufacturer’s instructions to measure the cytokines: IL-2, IL-4, IL-6, IL-10, TNF-α, INF-γ, and IL-17A. Fluorescence was measured using the BD Accuri^TM^ C6-Sample, flow cytometer (BD) and cytokine concentrations were calculated using a standard curve data of the FCAP Array software BD.

Results for all cytokines were calculated as the cytokine value of the rAg85A stimulated sample minus that of the non-stimulated sample.

### Enzyme*-*Linked Immunosorbent Assay (ELISA) for Anti-Ag85A Antibodies

Serum and bronchoalveolar lavage (BAL) antibodies against Ag85A were determined by ELISA. For serum: blood samples were taken from the animal’s temporal plexus prior to the first immunization and after mice were sacrificed. The BAL was performed according to a previously described technique (Medina et al., 2008).

The ELISA technique was performed in triplicate for each animal. Microliter plates were coated overnight at 4°C with 100 μL of rAg85A (2.5 μg/mL) in sodium carbonate/bicarbonate buffer (pH 9.6). Serum samples were diluted 1:25 (previously titrated) with PBS containing 0.05% (v/v) of Tween 20 (PBS-T). BAL samples were not diluted. After incubation with the samples for 2 h at 37°C, a horseradish peroxidase (HRP) conjugated goat anti-mouse IgG (1/2.500 in PBS-T) or IgA (1/8.000 in PBS-T) were added and incubated at 37°C for 2 h. Orthophenyldiamine (OPD), 1 mg/mL, was used for color development as an indicator. Absorbance was measured at 493 nm using a Bio-Rad Model 450 Microplate Reader.

### Statistical Analysis

In this study two individual experiments were performed, five animals were used in each group for each individual protocol and each animal was analyzed individually. Statistical analyses were performed using one-way analysis of variance (ANOVA) and the non-parametric Kruskal–Wallis test: Dunn’s multiple comparisons test, which is a post-test for multiple comparisons of the difference between the groups. No significant differences were observed between the two individual experiments performed. The data was expressed as mean ± standard deviation (SD) and *P*-values < 0.05 were taken as statistically significant. All analyses were performed using the Graph Pad Prism 6.0 statistical software.

## Results

### Recombinant Strain of *L. lactis*: *L. lactis* FnBPA^+^ (pValac:*Ag85A*)

The Ag85A ORF (1017 bp) (Gen Bank number 886132) was successfully cloned into the pValac vector (**Table 1**). This vector consists of an eukaryotic unit containing the cytomegalovirus promoter (pCMV) and the polyadenylation signal of the bovine growth hormone (BGH), and a prokaryotic portion containing the RepA/RepC replication origins for *L. lactis* and OriColE1 replication origin for *E. coli* and, as well as the chloramphenicol (Cm) resistance gene. The ORF of Ag85A was located between the pCMV and BGH polyA tail, as required for gene expression by eukaryotic cells. The construction of the pValac:*Ag85A* vector was confirmed by PCR, digestion, and sequencing. The invasive *L. lactis* FnBPA^+^ strain was then transformed with the pValac:*Ag85A* plasmid resulting in the recombinant invasive *L. lactis* FnBPA^+^ (pValac:*Ag85A*) strain.

### Eukaryotic Cells Can Express Ag85A Antigen

**FIGURE 2:**
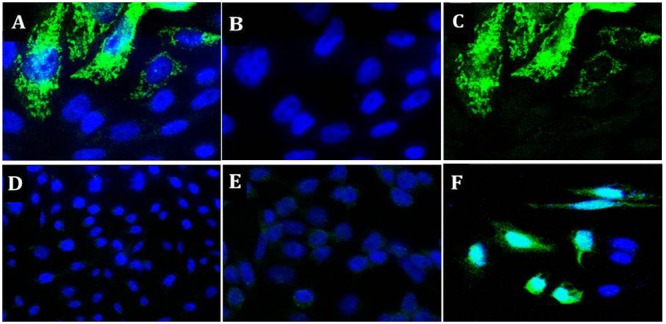
Expression of Ag85A protein by CHO cells transfected with the pValac:*Ag85A* vector: **(A)** image of CHO cells labeled with primary (Mab DT-17/4) and secondary [goat anti-mouse IgG (H+L)] antibodies, this is a merged image of images **(B,C), (B)** 4,6′-diamidino-2-phenylindole (DAPI) staining of the nuclei image, demonstrating intact nuclei, **(C)** Alexa 488 demonstrating Ag85A protein, **(D)** negative control: non-transfected CHO cells, **(E)** negative control: non-transfected CHO cells labeled with primary (Mab DT-17/4) and secondary [goat anti-mouse IgG (H+L)] antibodies, and **(F)** positive control: CHO cells transfected with pValac:*gfp*, this image is merged DAPI and GFP. 2D Images (x-y) are acquired in both depth (z-stack) using a Zeiss LSM 510 META inverted confocal laser-scanning microscope. **(A–C)** With 63X objective **(D–F)** 40 X objective.

Two independent assays confirmed the plasmid pValac:*Ag85A* functionality. Confocal microscopy analysis (after incubating the transfected CHO cells with specific mouse anti-Ag85A antibodies and a goat anti-mouse antibody labeled with Alexa 488), green fluorescence located in the cytoplasm specific for Ag85A protein expression was observed in cells transfected with the pValac:*Ag85A* vector (**Figure 2**). No Ag85A protein expression was observed neither in the CHO cells without transfection (without auto fluorescence by CHO cells), nor in the non-transfected cells labeled with the primary and secondary antibodies (negative controls). As a positive control, CHO cells transfected with pValac:*gfp* plasmid exhibited a high specific green fluorescence due to the expression of the GFP protein (**Figure 2**).

**FIGURE 3:**
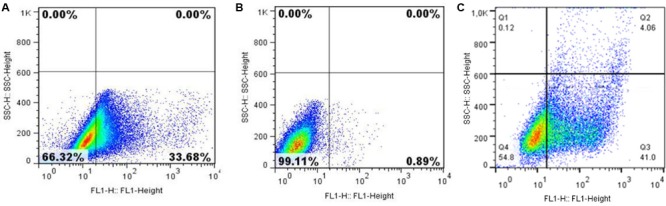
Expression of the Ag85A protein by CHO cells transfected with pValac:*Ag85A* vector. Single fluorescence-activated cell sorter (FACS). Dot Plots representing **(A)** transfected CHO cells labeled with primary (Mab DT-17/4) and secondary [goat anti-mouse IgG (H+L)] antibodies, **(B)** non-transfected CHO cells labeled with primary (Mab DT-17/4) and secondary [goat anti-mouse IgG (H+L)] antibodies (negative control), **(C)** pValac:*gfp* transfected cells (positive control). Images obtained using FlowJo software.

Flow cytometry showed approximately 33% of the transiently transfected cells with the pValac:*Ag85A* were able to express the Ag85A protein (**Figure 3**). In contrast, no expression was observed in the non-transfected cells. As expected, CHO cells transfected with the positive control pValac:*gfp*, showed a high level of green fluorescence because of GFP expression.

These results confirmed that the recombinant protein, rAg85A, was successfully expressed by eukaryotic cells, confirming the functionality of the pValac:*Ag85A* plasmid.

### Intranasal Administration of *L. lactis* FnBPA^+^ (pValac:*Ag85A*) Can Induce IFN-γ, TNF-α, and IL-6 Cytokines

**FIGURE 4:**
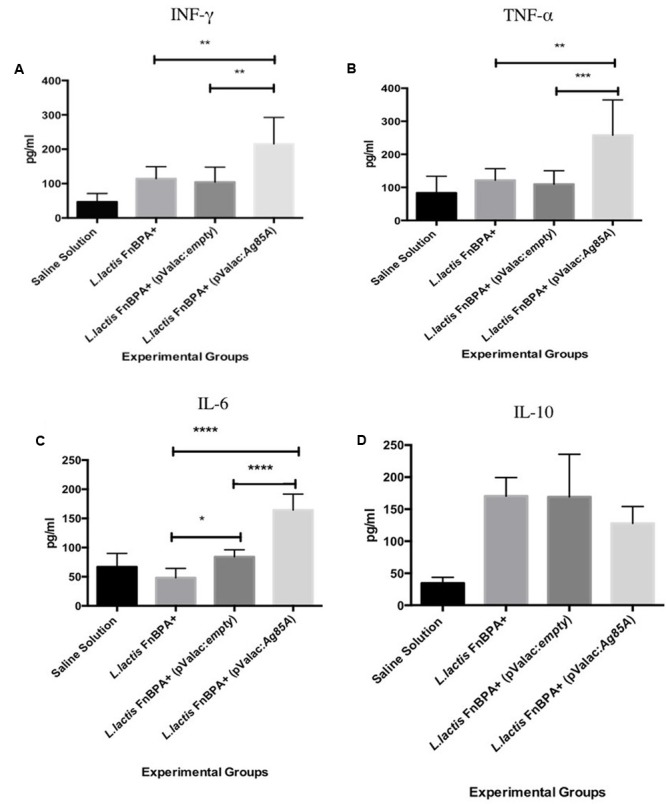
Production of cytokines from spleen cells stimulated with recombinant Ag85A. Production levels of **(A)** INF-γ, **(B)** IL6, **(C)** TNF-α, and **(D)** IL-10 in cultured supernatants of spleen cells. Experimental groups: Saline Solution (negative control), *Lactococcus lactis* FnBPA^+^(negative control), *L. lactis* FnBPA^+^ (pValac:*empty*), and *L. lactis* FnBPA (pValac:*Ag85A*). Data are shown as the mean ± SD. Two individual experiments were performed, five animals were used in each group for each individual protocol and each animal was analyzed individually. *p*-value: ^∗^*p* < 0.05, ^∗∗^*p* < 0.01, ^∗∗∗^*p* < 0.001, and ^∗∗∗∗^*p* < 0.0001.

Splenocytes of C57BL/6 mice immunized intranasally were re-stimulated, *in vitro* with the rAg85A protein and the culture supernatants were harvested to measure the cytokines production. The type 1 cytokine INF**-**γ was studied in this report due to its important function in host defense against *M. tuberculosis* infection. The results showed that the IFN-γ levels in the splenocyte supernatant in mice immunized with *L. lactis* FnBPA^+^ (pValac:*Ag85A*) was significantly increased compared with the level in *L. lactis* FnBPA^+^ (pValac:empty) group (*p* < 0.01) or in the *L. lactis* FnBPA^+^ group (*p* < 0.01) (**Figure 4A**). Also, it was possible to observe significant differences in other pro-inflammatory cytokines such as TNF-α and IL-6. The level of TNF-α in splenocyte supernatant in immunized mice with *L. lactis* FnBPA^+^ (pValac:*Ag85A*) was significantly increased compared with the level in *L. lactis* FnBPA^+^ (pValac:empty) group (p < 0.001) or in the *L. lactis* FnBPA^+^ group (*p* < 0.01) (**Figure 4B**); a significant increase in IL-6 levels in splenocyte supernatant of mice immunized with *L. lactis* FnBPA^+^ (pValac:*Ag85A*) (*p* < 0.0001) or those that received *L. lactis* FnBPA^+^ (*p* < 0.0001) was also observed (**Figure 4C**). No statistically significant differences were observed in IL-10 concentration between the analyzed groups (**Figure 4D**). The other cytokines analyzed (IL-2, IL-4, and IL-17A) were not detected in the supernatants of the spleen cells culture.

### *L. lactis* FnBPA^+^ (pValac:*Ag85A*) Can Induce a Systemic Immune Response

**FIGURE 5:**
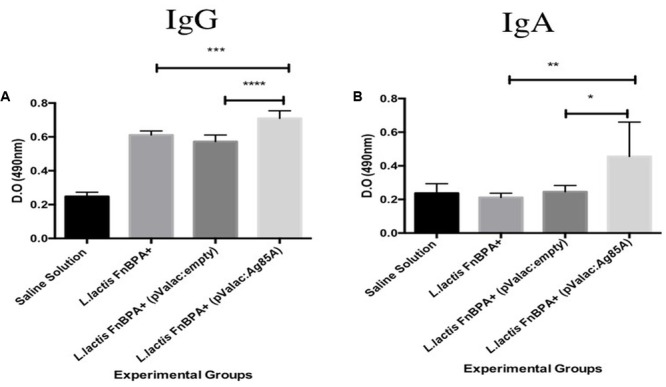
Antibodies production in serum of mice intranasally immunize. Production levels of **(A)** IgG anti-Ag85A and **(B)** IgA anti-Ag85A in serum of mice after 2 weeks of the last immunization. Experimental groups: Saline Solution (negative control), *L. lactis* FnBPA^+^(negative control), *L. lactis* FnBPA^+^ (pValac:*empty*), and *L. lactis* FnBPA (pValac:*Ag85A*). Data are shown as the mean ± SD. Two individual experiments were performed, five animals were used in each group for each individual protocol and each animal was analyzed individually. *p*-value: ^∗^*p* < 0.05, ^∗∗^*p* < 0.01, ^∗∗∗^, *p* < 0.001, and ^∗∗∗∗^*p* < 0.0001.

Immunoglobulins were measured in the serum of mice by ELISA test. The results showed that the anti-Ag85A IgG levels in serum of mice immunized with *L. lactis* FnBPA^+^(pValac:*Ag85A*) were significantly increased compared with those observed in the *L. lactis* FnBPA^+^ (pValac:*empty*) group (*p* < 0.0001) or in the *L. lactis* FnBPA^+^ group (*p* < 0.001) (**Figure 5A**). However, anti-Ag85A IgA levels in the serum of mice immunized with *L. lactis* FnBPA^+^ (pValac:*Ag85A*) was significantly increased compared to the animals in the *L. lactis* FnBPA^+^ (pValac:*empty*) group (*p* < 0.05) or in the *L. lactis* FnBPA^+^ group (*p* < 0.01) (**Figure 5B**). These differences demonstrated that the administration of *L. lactis* FnBPA^+^ (pValac:*Ag85A*) strain was able to induce a systemic immune response in *in vivo* assays.

### *L. lactis* FnBPA^+^ (pValac:*Ag85A*) Can Generate a Specific Mucosal Immune Response

**FIGURE 6:**
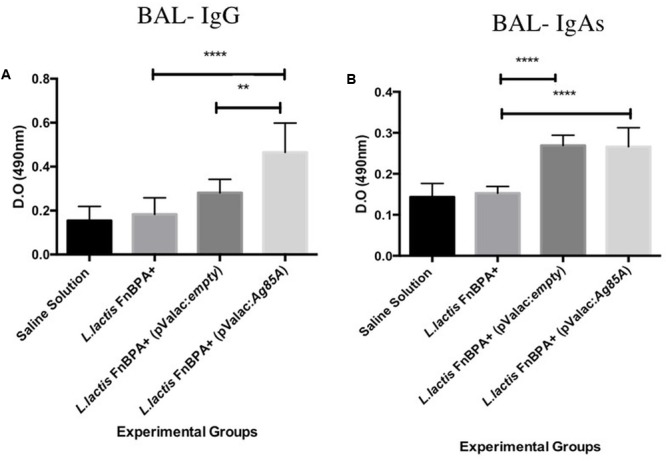
Antibodies production in bronchoalveolar lavage (BAL) of mice intranasally immunized. Production level of **(A)** IgG anti-Ag85A and **(B)** sIgA anti-Ag85A. Experimental groups: Saline Solution (negative control), *L. lactis* FnBPA^+^(negative control), *L. lactis* FnBPA^+^ (pValac:*empty*), and *L. lactis* FnBPA (pValac:*Ag85A*). Data are shown as the mean ± SD Two individual experiments were performed, five animals were used in each group for each individual protocol and each animal was analyzed individually. *p*-value: ^∗^*p* < 0.05, ^∗∗^*p* < 0.01, ^∗∗∗^*p* < 0.001, and ^∗∗∗∗^*p* < 0.0001.

In BAL samples, the levels of anti-Ag85A IgG in mice immunized with *L. lactis* FnBPA^+^(pValac:*Ag85A*) were significantly increased when they were compared with the levels of those immunized with *L. lactis* FnBPA^+^(pValac:*empty*) (*p* < 0.01) or in mice immunized with *L. lactis* FnBPA^+^ (*p* < 0.0001). However, anti-Ag85A IgAs levels did not show the same pattern since its levels in animals immunized with *L. lactis* FnBPA^+^ (pValac:*Ag85A*) did not show statistically significant differences with those that received *L. lactis* FnBPA^+^(pValac:*empty*) (**Figures 6A,B**).

## Discussion

Several vaccines strategies are currently being developed and many groups have studied the use of DNA vaccines using the intramuscular pathway (Dou et al., 2010; Yuan et al., 2012). However, this pathway requires very high quantities of the DNA vaccine (in the range of μg of DNA) and also an invasive pathway, which is not considered very effective in inducing the mucosal immune response, therefore reducing its capacity to prevent against infection at mucosal surfaces (Neutra and Kozlowski, 2006; Lycke, 2012).

Mucosal vaccination can induce mucosa-associated protection in the pathogenesis of airway disorders (Holmgren and Czerkinsky, 2005; Takizawa, 2005). The intranasal, as well as the bronchial epithelial cells are known to play a fundamental role in the airway defense mechanisms, and a critical role in mucosal immunity (Nizard et al., 2014) and are thus sites of interest for developing mucosal-based vaccines. Mucosal immune vaccination can activate both B and T cells which can drift to peripheral environmental, far distant from the one where the stimulation was induced (Meeusen, 2011).

Genetically modified LAB, have been used for heterologous proteins production (for a review, see Mancha-Agresti et al., 2015) and have also been used as delivery vehicles of molecules of interest including DNA, thus they are very promising candidates in the field of DNA vaccination. For this last application it was shown that both, native and invasive *L. la*ctis, can be used as *in vitro* and *in vivo* DNA delivery vehicles for plasmid transfer into mammalians cells (Guimaraes et al., 2006; Chatel et al., 2008; Innocentin et al., 2009; Almeida et al., 2014; Mancha-Agresti et al., 2016).

Recombinant invasive *L. lactis* are based on the concept of the expression of heterologous proteins called invasins, which allows and improves the internalization of bacteria inside eukaryotic cells. The recombinant *L. lactis* expressing the FnBPA protein from *S. aureus* (Que et al., 2001) were able to be internalized in Caco-2 cells (Innocentin et al., 2009). Another study using different invasive *L. lactis* (*S. aureus* FnBPA or *Listeria monocytogenes* mInlA) demonstrated that these recombinant strains are able to transfer DNA directly to mouse bone marrow-derived DCs, and also to invade a mono-layer of differentiated Caco-2 cells (de Azevedo et al., 2015) 100 times more efficiently than the wild-type lactococci.

Almeida et al. (2014) constructed another invasive strain using FnBPA invasin. They were able to increase the FnBPA expression (55%) when this invasin was expressed under the control of nisA promoter, enabling an increase of 3–4 times in invasiveness and internalization capacity in eukaryote cells .

The potential of invasive *L. lactis* as a delivery vector was recently shown using engineered *Lactococcus* carrying the pValac:*Esat-6*. The oral administration of this strain significantly increased INF-γ production by spleen cells, which indicated a systemic T helper 1 (Th1) response. This strain also significantly increased sIgA production in the colon, as well as in fecal extracts (Pereira et al., 2015). These results encourage the development of prime-boost strategies. Mice which received BCG prime/*L. lactis* FnBPA^+^ (pValac:*ESAT-6*) boosting showed significant increase of IL-17, IFN-γ, IL-6, and TNF-α cytokines produced by spleen cells. The BCG immune response was increased when boosted with recombinants invasive *L. lactis* (Pereira et al., 2017).

In this study, we used the intranasal route as an alternative to intramuscular and oral administration. The intranasal route has a great surface area, thin epithelium in the alveolar lung tissues, as well as extensive vascularization which enable an efficient delivery of the vaccine and decreases the dose required to induce protective immunity (Brian et al., 2007). This route has been shown to be effective in inducing effective protection against TB using lower doses (Rosada et al., 2008).

To this aim, we cloned Ag85A ORF of *M. tuberculosis* into pValac vector. Its functionality was confirmed by confocal microscopy and flow cytometry and was successfully transformed into *L. lactis* FnBPA^+^ (Invasive strain), producing the DNA vaccine *L. lactis* FnBPA^+^ (pValac:*Ag85A*). C57BL/6 mice received this DNA vaccine and its potential was evaluated.

Relating to the pro-inflammatory cytokines analyzed, statistically significant differences were shown in levels of INF-γ, IL-6, and TNF-α between groups that received *L. lactis* FnBPA^+^ carrying the Ag85A-coding vector compared with animals which received the empty vector, as well as animals which received the *L. lactis* FnBPA^+^ strains. As was previously reported, the immunity against TB includes different cytokines, cells groups, and mechanisms (Ottenhoff and Kaufmann, 2012; Andersen and Urdahl, 2015; Agger, 2016). INF-γ and TNF-α trigger the antimicrobial activity of macrophages (Flynn et al., 2001) and contribute to the recruitment of monocytes and granulocytes (Pfeiler and Klaenhammer, 2007). These cytokines (INF-γ, IL-6, and TNF-α) are important components of the protective response against TB (Andersen and Urdahl, 2015; Agger, 2016).

A similar approach using recombinant attenuated *aroA mutant of Salmonella typhimurium* carrying the *M. tuberculosis* Ag85A gene was performed by oral administration as well as through the intranasal route. The immune response in mice was evaluated and compared to the naked DNA approach. The assessment of IL-2 and INF-γ in the supernatant of re-estimulated splenocytes from immunized animals showed that the intranasal immunization offers better immunogenicity (Parida et al., 2005).

Acquired cell-mediated immune response identified by the production of type 1 cytokines is the typical component in host protection against mycobacterial infection (Cooper, 2009). Indeed, our results are indicative that the immunization with *L. lactis* FnBPA^+^(pValac:*Ag85A*) was capable of producing a Th1 systemic cellular immune response.

Many researches have tried to improve the existent BCG vaccine. In 2013, the intrapulmonary administration of purified recombinant Ag85A alone or combined with different adjuvants [unmethylated cytosine-phosphate-guanine motifs (CpG), the monophosphoryl lipid A of Salmonella minnesota (MPLA) or the B subunit of heat-labile enterotoxin of *E. coli* (LTB)] were tested (Todoroff et al., 2013). These authors showed that the administration of Ag85A with CpG or MPLA engendered polarized Th-1 immunity, desirable to protect against TB. Th-17 polarized immunity was generated with Ag85A plus MPLA administration. Although these authors have shown a good polarization Th1-or Th17 immunity, the pulmonary administration of the adjuvants did not increase the protection generated by Ag85A alone against a virulent challenge with *M. tuberculosis* (Todoroff et al., 2013). For this reason we proposed a different mode of administration of the antigen which would be produced locally and potentially be more effective as a vaccine.

In addition to T cell responses, antibodies provide a protective role in preventing mycobacterial infections. Neutralization of infectivity, inhibition of pathogen replication, phagocytosis, neutralization of toxins, antibody-dependent cellular cytotoxicity, and complement-mediated lysis of pathogens or of infected cells are some functions attributed to antibodies. Significant high levels of IgA and IgG anti-Ag85A were observed in the serum of C57BL/6 mice intranasally immunized with recombinant *L. lactis* FnBPA carrying the Ag85A-coding vector. Some recent reports have established that IgG antibodies could have protective effects in animal models of TB (Borrero et al., 2013; Olivares et al., 2013). Also, it is well established that the IgA antibodies are fundamental for the first line of defense against pathogens on the mucosal surface, and the idea of interconnected system, where the mucosal infection and/or immunization at one tissue sites is able to produce IgA production and protection at distant mucosal surface (Mestecky, 1987; Cerutti, 2008).

In the current study, it was shown that the intranasal vaccination was able to induce significant differences in IgG levels, in BAL fluid, between groups that received doses of *L. lactis* FnBPA^+^ carrying the Ag85A-coding vector compared to animals that received the strain carrying the empty vector, and to animals that received the *L. lactis* FnBPA^+^strains. However, no differences were observed in the IgAs levels between the experimental groups.

A recent study showed that higher titers of IgG against Ag85A are related with reduced risk of developing active disease in an infant case-control study (Fletcher et al., 2016). The existence of IgG antibody against Ag85A is related with decreased cavitation and a greater chance of sputum clearance of *M. tuberculosis* patients (Sánchez-Rodríguez et al., 2002). We were able to show statistical difference in the level of IgG anti-Ag85A between the animals immunized with the DNA vaccine and the negative controls.

The presence of IgA in nasal secretion is very important. A passive protection against *M. tuberculosis* challenge was reported when animals were intranasally immunized with specific IgA (Williams et al., 2004). After intranasal immunization, higher levels of IgA in the nasal secretion were observed suggesting that intranasal vaccination can induce local immune responses (Kiyono et al., 1991).

To summarize, the results show that *L. lactis* FnBPA (pValac:*Ag85A*) DNA vaccine elicits specific immune responses after intranasal administration. These results encourage us to test prime-boost vaccination strategies to obtain an amplified responses able to improve the immune protective respond here obtained and justify others researches using challenges with *M. tuberculosis* H37Rv.

## Ethics Statement

We here certify that the Protocol n 114/2015 related to the project entitle. Alternative strategy of DNA vaccination of infection and inflammatory diseases, under the supervisors of VA is in agreement with Ethical Principles in Animal experimentation, adopted by the Ethics Committee in Animal Experimentation (CETEA/UFMG, and was approved in 2015).

## Author Contributions

Conceptualization, PM-A and VA. Methodology, acquisition, and analysis, PM-A, CdC, MA, JdS, and VP. Investigation, PM-A, CdC, and JdS. Writing – Original Draft, PM-A. Writing – Review and Editing, PM-A, SL, JL, and VA. Funding Acquisition, VA. Supervision, VA. All the authors criticized and finally approved the final version of the manuscript.

## Conflict of Interest Statement

The authors declare that the research was conducted in the absence of any commercial or financial relationships that could be construed as a potential conflict of interest.
